# Associations of regular consumption of breakfast, lunch and dinner with Body Mass Index during adolescence: longitudinal findings by weight status among the Eating and Activity over Time 2010–2018 cohort

**DOI:** 10.1017/S1368980024000454

**Published:** 2024-02-22

**Authors:** Cynthia Y Yoon, Katherine R Arlinghaus, Tracey A Ledoux, Craig A Johnston, Nicole Larson, Dianne Neumark-Sztainer

**Affiliations:** 1 Department of Health and Human Performance, College of Liberal Arts and Social Sciences, University of Houston, 3875 Holman Street, Room 104, Houston, TX, USA; 2 Division of Epidemiology and Community Health, University of Minnesota School of Public Health, 1300 S 2nd St Suit 300, Minneapolis, MN, USA

**Keywords:** Breakfast, Lunch, Dinner, Weight gain, BMI, Longitudinal, Adolescents

## Abstract

**Objective:**

To examine how the associations between meal consumption and BMI over 8 years differ by weight status in a sample of adolescents.

**Design:**

Longitudinal, population-based study. Breakfast, lunch and dinner consumption and BMI were self-reported. Linear regressions were used to examine how the associations between meal consumption and BMI differed by weight status.

**Setting:**

Adolescents in the Minneapolis/St. Paul metropolitan area.

**Participants:**

Adolescents (*n* 1,471) were surveyed as part of the EAT 2010–2018 in 2009–2010 (M_age_ = 14·3 years) and 2017–2018 (M_age_ = 22·0 years).

**Results:**

The prevalence of regular breakfast, lunch and dinner consumption (≥ 5 times/week) ranged from 45 to 65 %, 75 to 89 % and 76 to 94 %, respectively, depending on weight status category. Among adolescents with a sex- and age-specific BMI < 15th percentile, regular consumptions of breakfast, lunch and dinner during adolescence were positively associated with BMI in emerging adulthood compared with irregular consumption of breakfast, lunch and dinner (<5 times/week) after adjustment for socio-demographic characteristics (*β* = 5·43, *β* = 5·39 and *β* = 6·46, respectively; all *P*-values <0·01). Among adolescents in the BMI 15–85th and 85–95th percentiles, regular consumptions of breakfast, lunch and dinner were positively associated with BMI but to a lesser extent (*P*-values <0·01). For participants with a BMI ≥ 95th percentile, regular consumptions of breakfast, lunch and dinner were positively associated with BMI, but the associations were not statistically significant (*P*-values > 0·05).

**Conclusions:**

The relationship between meal consumption during adolescence and BMI in emerging adulthood differs by adolescent weight status. Future studies should investigate underlying factors related to meal consumption routines and BMI.

Dietary habits that facilitate eating a nutrient-dense diet are important for fostering healthy growth and maintaining cardiometabolic health^(1,2)^. One often recommended dietary habit is to consume three nutritionally balanced meals (i.e. breakfast, lunch and dinner) on a daily basis (≥ 5 times/week). Among the three main meals, breakfast has been most extensively examined and cross-sectional and longitudinal studies have reported a positive association between the regular consumption of breakfast (≥ 5 times/week) and BMI among adolescents^(3,4)^ and young adults^(5,6)^. Fewer studies have investigated the associations of lunch and dinner with BMI^(7–9)^. Among the few studies examining the associations of lunch and dinner with BMI, a longitudinal study conducted among Japanese college students reported a positive association between dinner skipping and weight gain over time^(9)^. A cross-sectional study of Iranian adolescents^(8)^ and a longitudinal study of US youth^(7)^ have further reported an inverse association of regular consumption of dinner with BMI^(7,8)^.

The transition from adolescence to adulthood is widely recognised as a critical period during which eating patterns undergo significant changes^(10,11)^. Findings from a US nationally representative longitudinal study further document two distinct findings during this period: (1) a decrease in breakfast consumption and (2) excessive weight gain during the transitional period^(12)^. These findings suggest the need to examine associations between meal consumption and BMI trajectory over time. However, the lack of longitudinal studies examining associations between meal consumption and BMI during this developmental period precludes us from understanding the long-term effect of regular meal consumption on the BMI trajectory from adolescence to emerging adulthood (aged 18–25 years). This gap in the research is critical given that the rate of regular meal consumption is known to be the lowest during early adulthood^(13)^ and that the life stage of emerging adulthood is a unique period when independence and autonomy increase^(11)^ and young people begin to develop and practise their own eating habits^(10,14)^ that continue into later life^(11)^.

Another limitation of the current cross-sectional and longitudinal studies examining associations between meal consumption and BMI is that extant studies have been either inclusive of weight status, such as including participants regardless of their weight status without reporting weight-stratified results, or limited to youth with a sex- and age-specific BMI ≥ 10th percentile, or adults with a BMI ≥ 18·5 kg/m^2(5,6,9)^. Despite evidence that the relationship between consuming meals on a regular basis and future weight gain could differ by weight status, few studies have conducted stratified analyses^(15)^. Weight-stratified analyses could help to inform tailored recommendations in line with weight-related goals. For instance, among adolescents with a sex- and age-specific BMI < 5th percentile, intentional weight gain resulting in a higher BMI percentile could be seen as beneficial for their health^(16–18)^; among those with a sex- and age-specific BMI between the 5th and 85th percentiles, maintaining one’s current growth trajectory may be viewed as beneficial^(19)^; and among those with a sex- and age-specific BMI ≥ 85th percentile, a slowed rate of weight gain resulting in a lower BMI percentile might be viewed as beneficial^(20)^. Nevertheless, how the associations between regular meal consumption and BMI might differ as a function of weight status has not been fully explored. Examining how weight status affects the associations between regular consumption of meals and BMI may provide insight into the importance of regular meal consumption for weight management among individuals across all weight categories.

To address these issues, the primary aims of the present study were to (1) examine the prevalence of regular consumption of breakfast, lunch and dinner among adolescents by weight status; (2) examine how associations of regular consumption of breakfast, lunch and dinner with BMI over an 8-year follow-up period differ by weight status in a large population-based, ethnically/racially and socio-demographically diverse sample of adolescents and young adults. Thus far, several studies have suggested that regular breakfast consumption is associated with improved cardiometabolic health outcomes^(5,21)^. Other studies have further shown an inverse association between regular consumption of breakfast and BMI^(3–6)^. In a broader context, the American Academy of Pediatrics and Centers for Disease Control and Prevention recommend healthy eating to foster healthy growth and to maintain a healthy weight^(22,23)^. Taken together, we hypothesised that throughout 8 years of follow-up, adolescents in the < 15th percentile of sex- and age-specific BMI who regularly consumed meals would show a positive association with BMI compared with adolescents who irregularly consumed meals. Associations between regular meal consumption and BMI among adolescents between the 15th and 85th percentiles of sex- and age-specific BMI were examined on an exploratory basis with no hypotheses. Among adolescents in the ≥ 85th percentile of sex- and age-specific BMI, we hypothesised that regular meal consumption would be inversely associated with BMI over time.

## Methods

### Study design and population

EAT 2010–2018 (Eating and Activity over Time) is a population-based, longitudinal study of adolescents^(24,25)^. For EAT 2010, during the 2009–2010 academic year, 2,793 middle and senior high school students at twenty urban public schools in Minneapolis–St. Paul, Minnesota completed surveys including questions on their dietary intake and weight control behaviours and had their anthropometric measurements recorded by research staff. EAT 2018 was a follow-up study of EAT 2010 designed to track participants entering young adulthood in 2017–2018.

Among the 2,793 participants of the EAT 2010 study, invitations to participate in EAT 2018 were sent to 2,383 participants along with a two-dollar bill (410 participants were not followed due to lack of contact information and other reasons such as military deployment). To increase the response rate, non-responders received up to eight mailed reminders along with reminders via other modes of communication, including email, phone calls, text messages, social media and home visits. Participants were provided with financial compensation for their time to complete the EAT 2018 survey. Further information regarding the study design of EAT 2010–2018 is available in previous publications^(26,27)^.

The present study analysed data from 1,568 adolescents who participated in both EAT 2010 and EAT 2018, with the following exclusion criteria: missing data for breakfast (*n* 11), lunch (*n* 0) and dinner (*n* 1), missing self-reported BMI (*n* 6), implausible BMI (*n* 24) and one or more missing covariates (i.e. age, gender, ethnicity/race and socio-economic status) (*n* 55). The final analytic sample was composed of 1,471 participants (859 women, 601 men and eleven with a different gender identity, such as transgender, or non-binary).

To minimise potential response bias due to attrition that did not occur completely at random (i.e. non-responders were more likely than responders to be men, be nonwhite and have parents with low educational attainment at EAT 2010) and to extrapolate back to the original EAT 2010 school-based sample, inverse probability weighting^(28)^ was deployed. The weights for inverse probability weighting were calculated as the inverse of the estimated probability that an individual responded at both time points based on baseline age, sex, citizenship, ethnicity/race, socio-economic status, past-year dieting frequency and BMI. After weighting, there were no significant differences between the baseline and follow-up samples by demographic characteristics or weight status (*P* > 0·9). At baseline, the mean age of this sample was 14·3 years old (sd = 2·0). The weighted gender distribution of the sample was 58·5 % women, 40·8 % men and 0·7 % others, and the sample was diverse regarding ethnicity/race (24·1 % non-Hispanic White, 21·9 % African American, 17·1 % Hispanic, 22·5 % Asian and 14·3 % other ethnicity/race) and socio-economic status (36·7 % low, 21·8 % low to medium, 16·6 % medium, 16·1 % medium to high and 8·8 % high) (Table 1).

**Fig. 1:**
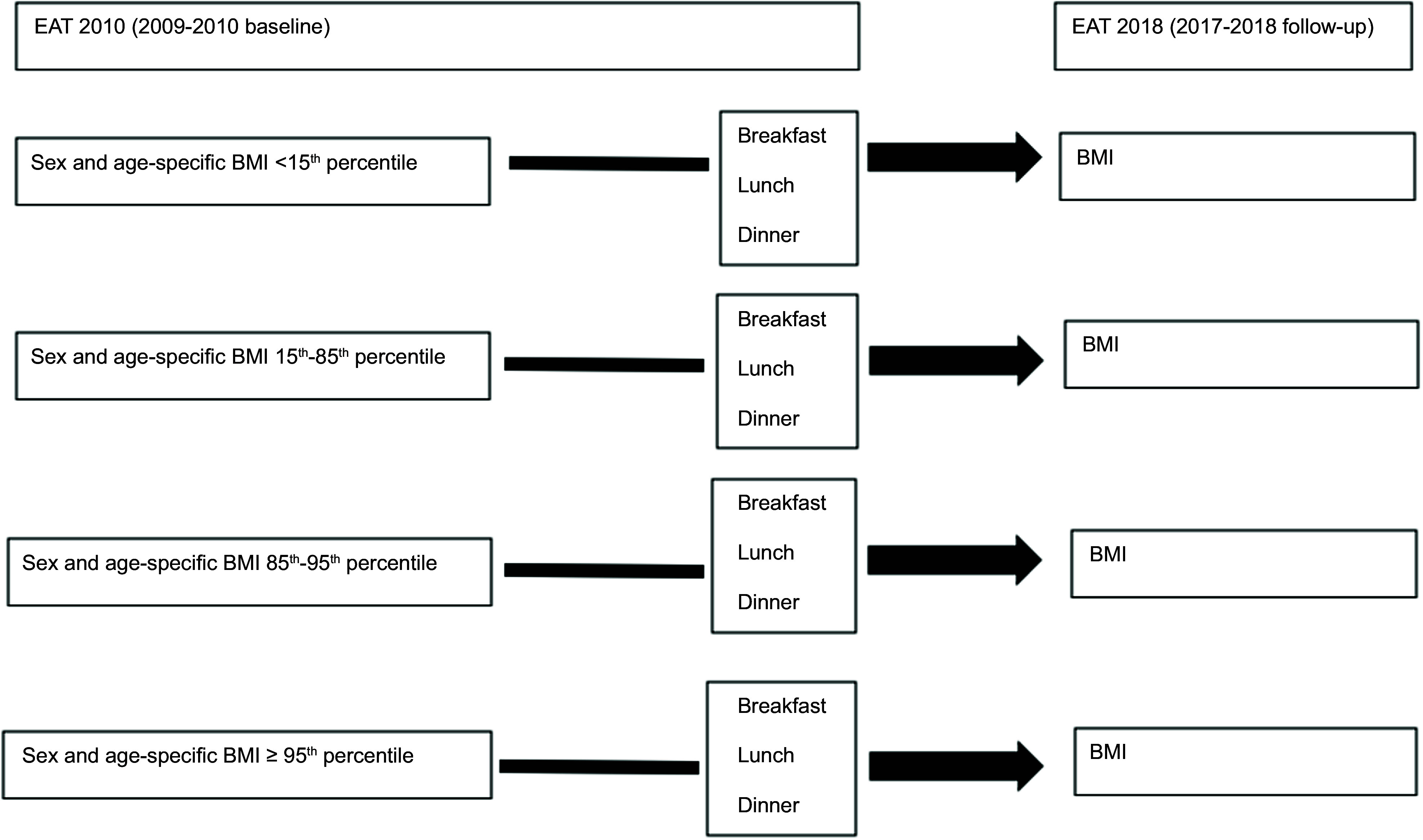
Flow chart of analyses of associations of frequency of breakfast, lunch and dinner intake with BMI by weight status

**Fig. 2:**
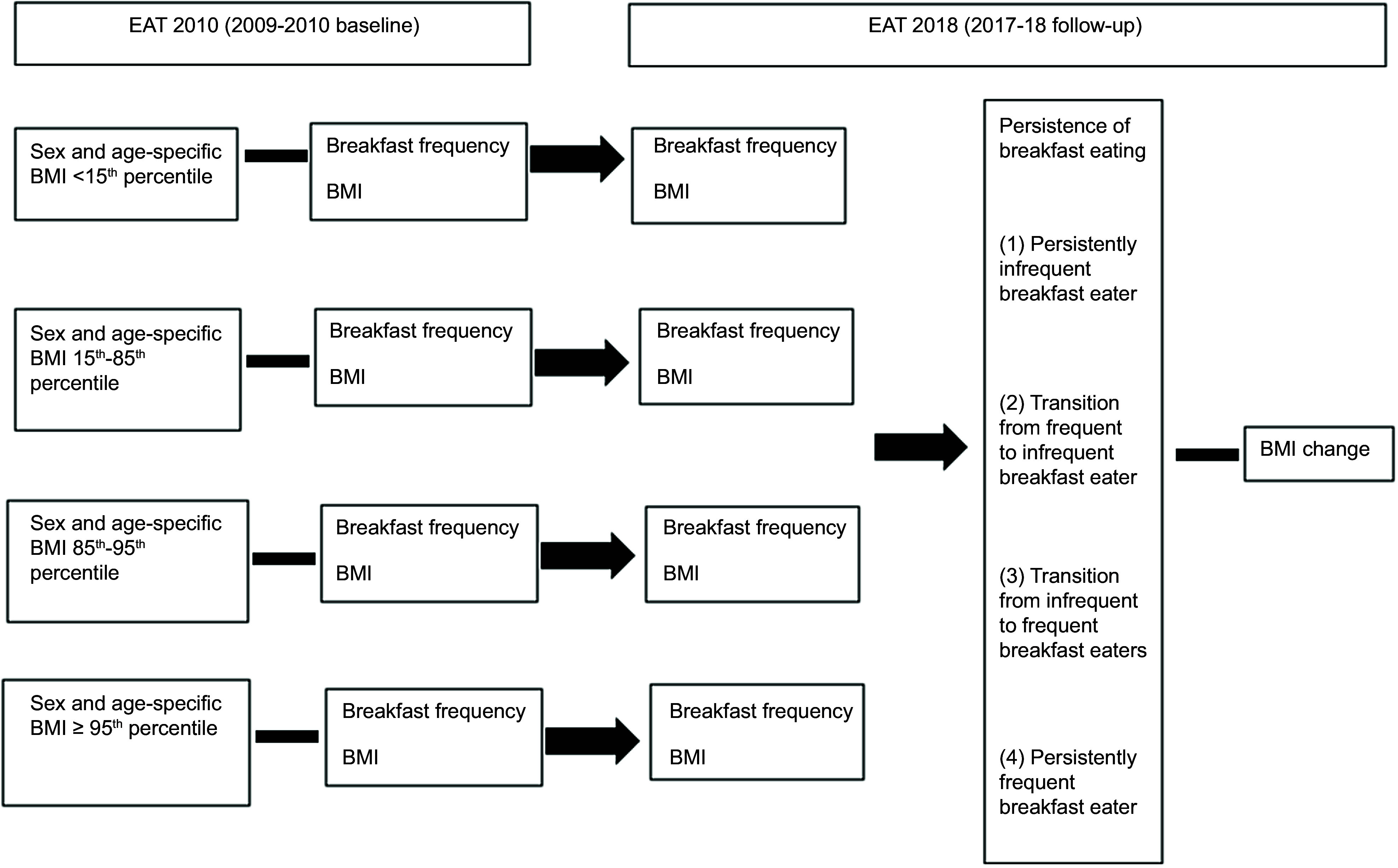
Flow chart of analyses of associations of persistency of breakfast intake with BMI change by weight status

The flow chart of the present study is shown in Figs. 1 and 2.

**Table 1:** Baseline characteristics of participants at EAT 2010 (*n* 1,471)

	Weight categories							
	Weight category 1 < 15th percentile of sex- and age-specific BMI (*n* 97)		Weight category 2 15–85th percentile of sex- and age-specific BMI (*n* 867)		Weight category 3 85–95th percentile of sex- and age-specific BMI (*n* 242)		Weight category 4 ≥ 95th percentile of sex- and age-specific BMI (*n* 265)	
	Mean or %	SD or *n*	Mean or %	SD or *n*	Mean or %	SD or *n*	Mean or %	SD or *n*
Age M ± sd	14·0	2·1	14·4	1·9	14·3	2·0	14·2	1·9
Sex column % (*n*)								
Male	43·3	42	37·3	323	38·4	93	54·0	143
Female	55·7	54	62·1	538	60·7	147	45·3	120
Others	1·0	1	0·7	6	0·8	2	0·8	2
Ethnicity/Race column % (*n*)								
White	27·8	27	28·0	243	14·9	36	19·3	51
Black/African American	21·7	21	20·8	180	27·7	67	19·3	52
Hispanics/Latino	15·5	15	15·9	138	19·4	47	20·0	53
Asian American	21·7	21	22·3	193	21·5	52	24·5	65
Native Hawaiian or other Pacific Islander	1·0	1	0·6	5	0·0	0	0·8	2
American Indian or Native American	3·1	3	3·0	26	3·7	9	7·9	21
Other	9·3	9	9·5	82	12·8	31	7·9	21
SES column % (*n*)								
Low	28·9	28	34·7	301	38·0	92	43·4	115
Low-medium	16·5	16	21·5	186	26·9	65	20·8	55
Medium	15·5	15	16·2	140	16·9	41	18·1	48
Medium-high	19·6	19	17·2	149	14·1	34	13·6	36
High	19·6	19	10·5	91	4·1	10	4·2	11
Breakfast column % (*n*) (how many days did you eat breakfast)								
Never	6·2	6	9·7	84	10·7	26	12·5	33
1–2 d/week	15·5	15	20·0	173	26·0	63	26·4	70
3–4 d/week	13·4	13	16·6	144	17·8	43	16·6	44
5–6 d/week	14·4	14	13·2	114	15·7	38	13·2	35
Everyday	50·5	49	40·6	352	29·8	72	31·3	83
Lunch column % (*n*) (how many days did you eat lunch)								
Never	2·1	2	2·2	19	3·3	8	1·1	3
1–2 d/week	2·1	2	5·4	47	9·9	24	7·6	20
3–4 d/week	7·2	7	10·4	90	11·6	28	10·9	29
5–6 d/week	13·4	13	13·0	13	14·5	35	14·3	38
Everyday	75·3	73	69·0	598	60·7	147	66·0	175
Dinner column % (*n*) (how many days did you eat dinner)								
Never	0·0	0	1·2	10	01·7	4	1·5	4
1–2 d/week	2·1	2	3·5	30	4·1	10	6·0	16
3–4 d/week	4·1	4	8·5	74	18·2	44	13·2	35
5–6 d/week	10·3	10	15·2	132	13·2	32	10·2	27
Everyday	83·5	81	71·6	621	62·8	152	69·1	183

Weight categories are based on BMI percentile at 2009–2010 (EAT 2010).

### Eating and Activity over Time survey development

Selected items from the EAT 2010 survey were retained in the EAT 2018 survey to ensure longitudinal comparisons. However, changes were made to account for societal trends and the developmental progression of participants from adolescence to young adulthood. To refine the EAT 2018 survey, feedback was obtained from three focus groups (*n* 29), and the revised version of the survey was reviewed by experts in nutrition, physical activity, body image and family relations. The EAT 2018 survey’s psychometric properties were examined using data from the full sample of participants who completed the EAT 2018 survey. The test–retest reliability coefficients for finalised survey items were obtained from a subgroup of 112 participants who completed the survey twice within a 3-week period.

### Measures

#### Regular consumption of breakfast

Breakfast consumption was assessed using a single item: ‘During the past week, how many days did you eat breakfast?’ The response options were ‘never’, ‘1–2 d’, ‘3–4 d’, ‘5–6 d’ and ‘every day’. Participants who responded ‘5–6 d’ or ‘every day’ were categorised as having regular breakfast consumption. All other respondents were classified as having irregular breakfast consumption (test–retest *r* = 0·77 at EAT 2018). The cut-off points used to dichotomise breakfast consumption were based on the cut-off points used in other studies^(3,29)^.

#### Regular lunch consumption

Lunch consumption was assessed using a single item: ‘During the past week, how many days did you eat lunch?’ The response options were ‘never’, ‘1–2 d’, ‘3–4 d’, ‘5–6 d’ and ‘every day’. Participants who responded ‘5–6 d’ or ‘every day’ were categorised as exhibiting regular lunch consumption. All other respondents were classified as exhibiting irregular lunch consumption (test–retest *r* = 0·60 at EAT 2018).

#### Regular dinner consumption

Dinner consumption was assessed using a single item: ‘During the past week, how many days did you eat dinner?’ The response options were ‘never’, ‘1–2 d’, ‘3–4 d’, ‘5–6 d’ and ‘every day’. Participants who responded ‘5–6 d’ or ‘every day’ were categorised as exhibiting regular dinner consumption. All other respondents were classified as exhibiting irregular dinner consumption (test–retest *r* = 0·76 at EAT 2018).

#### Persistence of breakfast over time

Based on breakfast consumption categories at baseline (2009–2010) and at follow-up (2017–2018), persistence of breakfast consumption was categorised into four groups: (1) irregular breakfast consumption at baseline and follow-up (i.e. irregular breakfast over time); (2) irregular breakfast consumption at follow-up only (i.e. transition from regular to irregular breakfast consumption); (3) regular consumption at follow-up only (i.e. transition from irregular to regular breakfast consumption) and (4) regular breakfast consumption at baseline and follow-up (i.e. regular breakfast over time). Persistence of lunch and dinner was not examined because lunch and dinner were only assessed at baseline (2009–2010) and not in 2017–2018.

#### BMI

BMI was calculated primarily using self-reported height and weight. For participants missing self-reported height and weight at baseline, a height and weight measured by a trained research staff member in a private area at each school was used to calculate BMI (test–retest reliability = 0·99, 0·97 and 0·98 for self-reported weight, height and BMI, respectively, at EAT 2018). High correlations were reported between measured and self-reported in the entire sample of male and female participants^(30)^. In this study, baseline BMI at EAT 2010 was used to classify weight status into four different categories: (1) weight category 1: <15th percentile of sex- and age-specific BMI; (2) weight category 2: between the 15th and 85th percentiles of sex- and age-specific BMI; (3) weight category 3: 85–95th percentile of sex- and age-specific BMI and (4) weight category 4: ≥ 95th percentile of sex- and age-specific BMI. Sex- and age-specific BMI percentiles were used to account for the body composition and growth patterns relevant to adolescents’ sex and age. Sex- and age-specific BMI percentile cut-points were selected based on earlier studies drawn from the EAT 2010–2018 study^(31)^ (weight status % agreement = 89 % at EAT 2018). BMI at EAT 2018 was analysed as a continuous variable to track the BMI trajectory.

#### Covariates

Variables that were considered potential confounders of the associations between meal consumption and BMI were included in the models if they met the following criteria: (1) variables associated with BMI, (2) variables associated with meal consumption (i.e. variables unequally distributed among meal consumption frequency) and (3) variables not part of the causal pathway between meal consumption and BMI^(32)^. Variables considered potential confounders for this study included age (based on the date of birth), gender, ethnicity/race and socio-economic status, which were self-reported in the EAT 2010 survey. A classification and regression tree-based algorithm was used to determine socio-economic status. Socio-economic status was primarily based on the highest level of educational attainment of either parent. Other factors used to determine socio-economic status included family eligibility for public assistance, eligibility for free or reduced-cost school meals and maternal and paternal employment status (test–retest *r* = 0·90)^(33)^.

### Statistical analysis

Participant characteristics are presented as the mean (sd) or % frequency. The associations of baseline meal intake (i.e. breakfast, lunch and dinner) with BMI over 8 years were examined using linear regressions, after ensuring that the assumptions for linear regressions were met. All models were adjusted for age, gender, ethnicity/race and socio-economic status, which could be confounders of the association between meal consumption and BMI. Considering that weight status at baseline might moderate the association between regular meal consumption and BMI, interactions between weight status at baseline (i.e. BMI percentile) and regular meal consumption (i.e. breakfast, lunch and dinner) with respect to BMI were examined. Because of the significant interaction terms between meal (i.e. breakfast, lunch and dinner) consumption and weight status (i.e. sex- and age-specific BMI percentiles) on BMI (*P*-value <0·01), weight status-stratified results for all findings are presented. All models were weighted by non-response propensity to reflect the EAT 2010 sample population. Given the growing criticism of null hypothesis significance testing^(34)^, in this study, we emphasise effect estimation in reporting our results. The statistical significance level was set at 0·05 for all analyses performed in this study. Statistical analyses were conducted using SAS software, version 9.4 (SAS Institute Inc.).

## Results

### Prevalence of regular consumption of breakfast, lunch and dinner by weight status

**Fig. 3:**
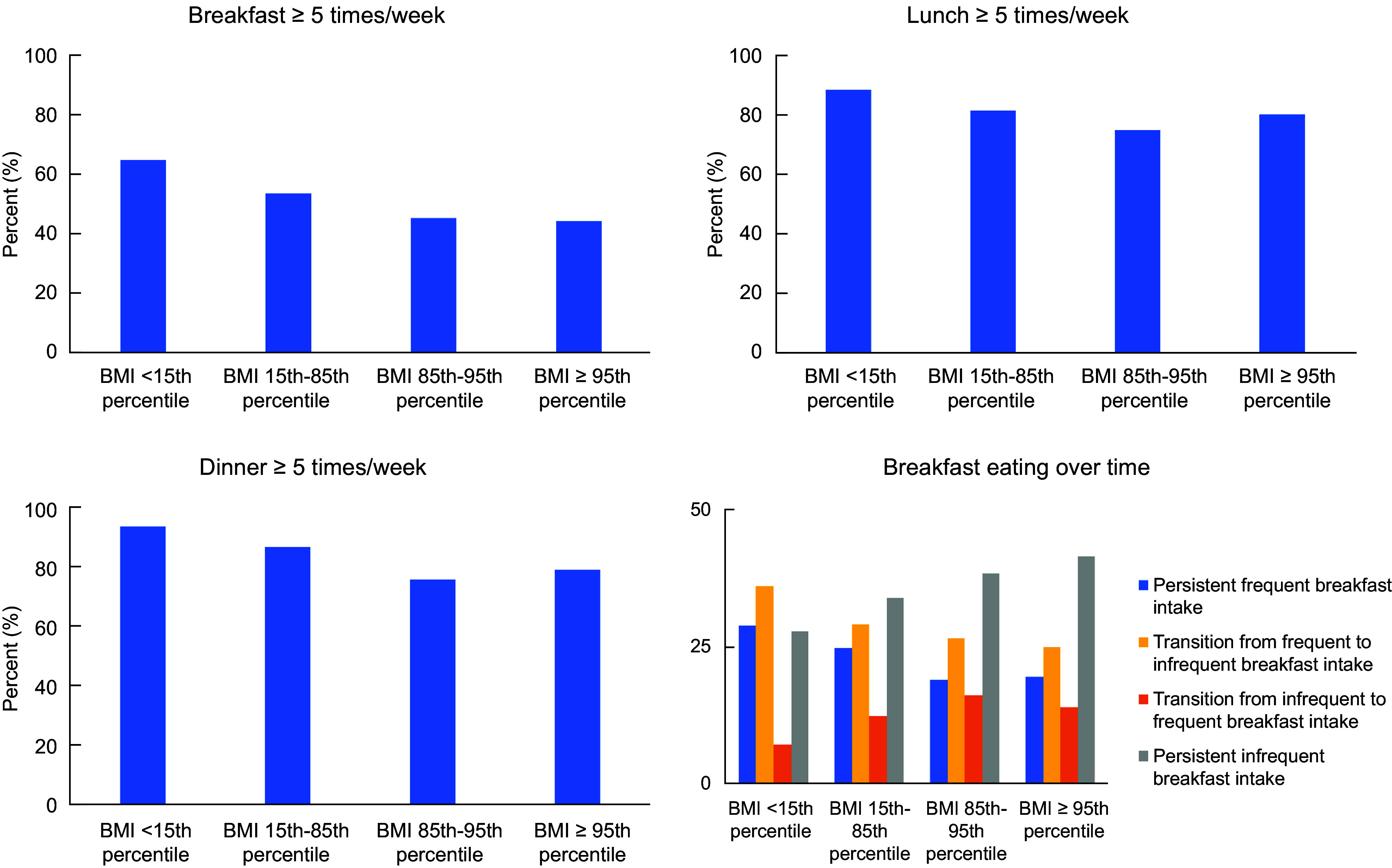
Prevalence of regular and persistent consumption of breakfast, lunch and dinner (*N* 1471). Sex- and age-specific BMI < 15th percentile, *n* 97; sex- and age-specific BMI in 15–85th percentile, *n* 867; sex- and age-specific BMI 85–95th percentile, *n* 242; sex- and age-specific BMI ≥ 95th percentile, *n* 265. Regular eaters: eating meal ≥ 5d/week

In this study, regular breakfast consumption (≥ 5 d/past week) was reported by approximately 65 % of the adolescents below the 15th percentile of sex- and age-specific BMI (weight category 1), 54 % of adolescents between the 15th and 85th percentiles of BMI (weight category 2), 45 % of adolescents between the 85th and 95th percentiles of BMI (weight category 3) and 45 % of adolescents above the 95th percentile of BMI (weight category 4). The prevalence of regular lunch consumption was 89 %, 82 %, 75 % and 81 % in weight categories 1–4, respectively. Regular dinner consumption was reported by 94 %, 87 %, 76 % and 79 % of adolescents in weight categories 1–4, respectively (Fig. 3). Details about the persistence of breakfast consumption over time are presented for each weight category in Fig. 3.

### BMI trajectories by weight status and meal type

Informed by the statistically significant interaction terms between meal consumption and weight status (i.e. sex- and age-specific BMI percentiles) in relation to BMI (*P*-value for the interaction term <0·01), the detailed associations between regular meal consumption (i.e. breakfast, lunch and dinner) and BMI across the 8-year follow-up for each weight category are presented below.

#### Weight category 1 (<15th percentile of sex- and age-specific BMI)

After 8 years of follow-up, regular consumption of breakfast at baseline was associated with a higher mean BMI than irregular breakfast consumption (BMI difference= 5·43 ± 0·62 kg/m^2^ in 2017–2018). Similar patterns were found for lunch and dinner; regular consumption of lunch and dinner was associated with a higher mean BMI than irregular consumption of meals (BMI difference = 5·39 ± 0·63 and 6·46 ± 0·72 kg/m^2^ for lunch and dinner, respectively, in 2017–2018) (Table 2).

**Table 2:** Irregular and regular breakfast, lunch and dinner consumption and mean BMI in 2017–18 (*n* 1471)

		Weight category 1 <15th percentile of sex- and age-specific BMI (*n* 97)					Weight category 2 15–85th percentiles of sex- and age-specific BMI (*n* 867)					Weight category 3 85–95th percentiles of sex- and age-specific BMI (*n* 242)					Weight category 4 ≥ 95th percentile of sex- and age-specific BMI (*n* 265)				
		*n*	*β*	se	*R* ^2^	*P* value	*n*	*β*	se	*R* ^2^	*P* value	*n*	*β*	se	*R* ^2^	*P* value	*n*	*β*	se	*R* ^2^	*P* value
B	Irregular (ref)	34	0·00 (ref)				401	0·00 (ref)				132	0·00 (ref)				147	0·00 (ref)			
	Regular	63	5·43	0·62	0·47	<0·01	466	3·68	0·26	0·30	<0·01	110	4·34	0·62	0·31	<0·01	118	0·73	0·82	0·15	0·38
L	Irregular (ref)	11	0·00 (ref)				156	0·00 (ref)				60	0·00 (ref)				52	0·00 (ref)			
	Regular	86	5·39	0·63	0·45	<0·01	711	3·78	0·25	0·30	<0·01	182	4·74	0·53	0·31	<0·01	213	1·10	0·76	0·15	0·15
D	Irregular (ref)	6	0·00 (ref)				114	0·00 (ref)				58	0·00 (ref)				55	0·00 (ref)			
	Regular	91	6·46	0·72	0·50	<0·01	753	4·04	0·27	0·30	<0·01	184	4·39	0·52	0·31	<0·01	210	1·37	0·78	0·15	0·08

B, breakfast; L, lunch; D, dinner.

Regular eaters: eating meal ≥ 5 d/week.

Irregular eaters: eating meal <5 d/week.

Models adjusted for socio-demographic characteristic (age, ethnicity/race, gender and socio-economic status).

Regarding the persistence of breakfast consumption over 8 years, there was no significant difference in mean BMI change across groups compared with persistence irregular breakfast intake (all *P* values >0·05) (Table 3).

**Table 3:** Persistence of breakfast eating and BMI change between 2010 and 2018 (*n* 1471)

	Weight category 1 <15th percentile of sex- and age-specific BMI					Weight category 2 15–85th percentile of sex- and age-specific BMI					Weight category 3 85–95th percentile of sex- and age-specific BMI					Weight category 4 ≥ 95th percentile of sex- and age-specific BMI				
	*n*	*β*	se	*R* ^2^	*P* value	*n*	*β*	se	*R* ^2^	*P* value	*n*	*β*	se	*R* ^2^	*P* value	*n*	*β*	se	*R* ^2^	*P* value
Persistence irregular breakfast intake (ref)	27	0·00 (ref)				294	0·00 (ref)				93	0·00 (ref)				93	0·00 (ref)			
Transition from regular to irregular breakfast intake	35	1·65	1·20	0·04	0·17	252	–0·04	0·41	0·04	0·91	64	0·23	1·08	0·04	0·83	64	–0·83	1·31	0·04	0·53
Transition from irregular to regular breakfast intake	7	0·60	2·06	0·04	0·77	107	–0·74	0·53	0·05	0·16	39	–0·26	1·33	0·05	0·84	39	–2·35	1·56	0·05	0·13
Persistence regular breakfast intake	28	0·39	1·33	0·04	0·77	214	–1·02	0·42	0·04	0·01	46	–2·18	1·18	0·04	0·07	46	–2·05	1·43	0·04	0·15

Adjusted for socio-demographics (age, ethnicity/race, gender and socio-economic status).

In each weight category, BMI change sharing the same superscript indicates BMI changes are not different from each other (*P* < 0·05).

#### Weight category 2 (15th–85th percentiles of sex- and age-specific BMI)

After 8 years of follow-up, adolescents who regularly consumed breakfast, lunch and dinner had a higher mean BMI than those who irregularly consumed breakfast at baseline (BMI difference= 3·68 ± 0·26, 3·78 ± 0·25 and 4·04 ± 0·27 kg/m^2^ for breakfast, lunch and dinner, respectively, in 2017–2018 Table 2).

Persistent regular consumption of breakfast over 8 years was associated with a decrease in mean BMI compared with the consistent irregular consumption of breakfast. Among those who transitioned from regular to irregular breakfast consumption or from irregular to regular breakfast consumption over 8 years of follow-up, the mean BMI did not differ from that of consistently irregular breakfast eaters (Table 3).

#### Weight category 3 (85th–95th percentiles of sex- and age-specific BMI)

After 8 years of follow-up, regular consumption of breakfast and dinner at baseline was associated with a greater mean BMI than irregular consumption of meals (mean BMI difference= 4·34 ± 0·62, 4·74 ± 0·53 and 4·39 ± 0·52 kg/m^2^ for breakfast, lunch and dinner, respectively, in 2017–2018, *P* value < 0·01). (Table 2).

Persistence of regular breakfast consumption over 8 years was associated with a decrease in BMI compared with persistently and irregularly consuming breakfast. For individuals whose breakfast consumption frequency fluctuated over 8 years (i.e. transitioned from regular to irregular consumption and vice versa), the mean BMI change did not differ from that of persistently irregular breakfast eaters (Table 3).

#### Weight category 4 (≥ 95th percentile of sex- and age-specific BMI)

After 8 years of follow-up, both regularly consuming breakfast and lunch at baseline were associated with a modestly but not statistically significantly higher mean BMI compared with irregularly consuming the respective meals (BMI difference = 0·73 ± 0·82, 1·10 ± 0·76, 1·37 ± 0·78 for breakfast, lunch and dinner in 2017–2018, *P* values ≥ 0·08) (Table 2).

Regarding the persistence of breakfast consumption over 8 years, participants whose breakfast intake frequency changed, regardless of the direction, had a lower mean BMI than those who remained persistent breakfast eaters (Table 3).

## Discussion

This study of adolescents and the transition to emerging adulthood examined (1) the prevalence of regular breakfast, lunch and dinner consumption and the persistence of breakfast consumption over time; (2) associations of the regular consumption of breakfast, lunch and dinner with BMI over 8 years of follow-up and (3) differences by weight status and meal type in the relationship between regular consumption of meals and BMI over 8 years of follow-up. In this study, among adolescents, regular consumption of dinner (≥ 5 times/week) was most consistent across weight status, followed by lunch and breakfast. Regular consumption of each meal was longitudinally associated with BMI, but this association differed based on weight status, meal and persistence of meal consumption pattern over time.

The prevalence of regular meal consumption among adolescents in this study ranged from 44 % to 95 %, depending on the meal, which was consistent with that reported in other population-based longitudinal studies of US adolescents^(3)^ and cross-sectional studies of Canadian adults^(35)^. We further expand the literature by documenting that the prevalence of regular meal consumption differs by weight status. In this study, adolescents below the 15th percentile of sex- and age-specific BMI had the highest rate of regular consumption of breakfast, lunch and dinner, and persistence of regular breakfast consumption compared with those in the other weight categories (≥ 15th percentile of sex- and age-specific BMI). The findings from the present study may suggest that it may be informative for future studies to further explore the underlying weight-specific factors related to regular consumption of breakfast. For example, in addition to cross-sectional and longitudinal studies of US adolescents that suggest body dissatisfaction, weight-related teasing from peers or family members, which can lead to distress and less frequent meal consumption^(36–38)^, future studies should explore whether parental pressure to eat regularly may serve as a weight-specific factor related to regular consumption of meals among adolescents below the 15th percentile of sex-and age-specific BMI.

Socio-ecological theory^(39)^ further provides a framework for examining how community and societal factors, such as belonging and relationships in different contexts such as family, peers, community and society, may be related to meal intake during adolescence. Examining the sense of belongingness to a social network and social isolation during adolescence as potential predictors of meal consumption is crucial, as adolescence is a developmental period when individuals are exposed to various physical and social environments, have opportunities to interact with different individuals and when peer pressure peaks, which may influence their eating behaviours. Therefore, future research should consider examining community and societal factors that may impact lunch and dinner consumption during adolescence.

Regarding associations between the regular consumption of meals and BMI, the parameter estimate differed based on weight status and type of meal. As expected, after 8 years of follow-up, among adolescents below the 15th percentile of sex- and age-specific BMI, regular consumption of breakfast was associated with a higher mean BMI than irregular consumption of breakfast. Although it is plausible that the higher mean BMI after 8 years of follow-up may indicate healthy weight gain, caution is required when interpreting the results given that the nutrient content or the quality of breakfast was not assessed in this study. We further report that regular consumption of lunch and dinner was also associated with a higher mean BMI in emerging adulthood; however, the mean BMI of regular meal consumers was not in a range associated with increased cardiometabolic risk (as defined by the Centers for Disease Control and Prevention)^(40)^.

Among adolescents with sex- and age-specific BMI in the 15th–85th percentiles, regular consumption of breakfast was associated with a higher BMI than the irregular consumption of breakfast after 8 years of follow-up. These findings were similar among adolescents with sex- and age-specific BMI in the 85th–95th percentile. These findings align with a randomised controlled study of adolescents that suggests that breakfast consumption may lead to greater weight gain and caloric consumption^(41)^. However, these results are in contrast to prior research indicating an inverse relationship between daily breakfast consumption and BMI in US adolescents^(3)^ and adults^(5)^, as well as to longitudinal studies of Japanese college students which had a follow-up period of 3 years^(9)^ and US health professionals, which had a follow-up period of 10 years^(6)^. Disparate findings may have occurred from the different study designs and the lack of extant studies that explored associations by weight status and meal type. The disparate findings indicate the importance of future research in this area of associations of meal consumption with BMI that considers baseline weight status, meal type, context of the meal and follow-up period.

Regarding lunch and dinner consumption, the present study’s finding of suggestive positive associations of lunch and dinner consumption with BMI after an 8-year follow-up period among adolescents within sex- and age-specific BMI in the 15–85th and 85–95th percentiles contrasts with the results from a few cross-sectional studies involving Iranian adolescents and Spanish adults^(8,42)^ and one longitudinal Japanese study of college students^(9)^, which reported an inverse association of lunch and dinner consumption with BMI. Additionally, the inconsistent findings between the present study of an 8-year follow-up period and the longitudinal study of Japanese college students with a 3-year follow-up study suggest that dinner consumption may have a short-term inverse association with BMI but may be positively associated with a higher BMI in the long term. The discrepancy between the studies may have partially resulted from the difference in the context of meals, mealtime environmental factors and meal composition. For example, in the present study, participants consisted of adolescents. As a result, it is probable that in the present study, meals were predominantly consumed at home. In contrast, previous studies involved young college students or adults who typically prepare and consume their meals independently, or with friends, or consume meals outside home.

Regarding adolescents above the 95th percentile of sex- and age-specific BMI, notably, and in contrast to our hypothesis, regular consumption of breakfast, lunch and dinner was each associated with a higher mean BMI after 8 years of follow-up compared with the irregular consumption of dinner, although not all findings were not statistically significant.

Although the food items explicitly consumed or what constituted a meal were not assessed, the higher BMI among adolescents who regularly consume dinner may suggest that adolescents with a sex- and age-specific BMI > 95th percentile who self-reported regular consumption of dinner might routinely skip breakfast and lunch as a weight loss strategy and consume larger portion sizes and select foods that are high in calories with fewer nutrients for dinner^(43)^. Alternatively, these adolescents might be less attuned to internal hunger cues, resulting in poor intuitive eating skills. Additionally, although the timing of dinner was not assessed in this study, regular consumption of dinner could have repeatedly occurred late at night (i.e. close to bedtime) when ghrelin levels are higher compared with the morning, which might be associated with a stronger appetite and craving for energy-dense foods^(44)^. Furthermore, the positive association of regular dinner consumption with a higher BMI reported in this study might be related to erratic sleep/wake patterns (e.g. short duration and poor quality of sleep and extended hours of wakefulness), which may lead to inappropriate internal circadian timing of food intake^(45,46)^, resulting in excessive weight gain^(47)^. Given that an inverse association was reported for the consumption of other meals, additional studies should be performed to examine why such a positive association between regular consumption of dinner and greater BMI over time was observed exclusively among young people in this weight category.

We further report that among adolescents between the 15th and 85th percentiles and between the 85th and 95th percentiles of sex- and age-specific BMI, persistence of regular consumption of breakfast at two time points over 8 years was associated with a decrease in BMI compared with those with a consistent irregular consumption of breakfast. Such findings may imply that consistent breakfast consumption over an extended period may lead to greater satiety reducing hunger throughout the day, and may be effective in managing weight over time^(48)^.

Overall, our study has several strengths. We expanded the extant literature by including a broader spectrum of meals, including lunch and dinner, which have not been extensively studied. This study also included adolescents in the <15th percentile of sex- and age-specific BMI, a group that has been frequently underrepresented in the literature on meal skipping. The inclusion of individuals across the entire BMI spectrum allowed us to stratify the results based on weight status and to demonstrate the differences in terms of direction and magnitude. Additionally, our study had a substantial follow-up time of 8 years, allowing us to track the association of lunch and dinner consumption with BMI from adolescence (aged 11–18 years) to emerging adulthood (age 18–30 years). This study’s inclusion of an ethnically/racially diverse sample of adolescents adds to the current literature on meal consumption, which has mainly focused on non-Hispanic whites^(3,5,6)^ in the USA. To further increase the generalisability of our findings, future studies should replicate these results by sampling participants from a wider range of socio-economic backgrounds.

Several limitations should also be noted. First, meal consumption was self-reported. However, the test–retest reliability for meal consumption was high (*r* = 0·77, *r* = 0·60 and *r* = 0·76 for breakfast, lunch and dinner, respectively in the EAT 2018 cohort). Additionally, BMI was calculated primarily using self-reported heights and weights, which might have biased the association towards the null due to non-differential misclassification. However, Project EAT, which is the original dataset of this current study, tracked BMI from adolescence to adulthood and reported that emerging adults’ self-reports of height and weight are highly correlated with measured height and weight, suggesting validity for the assessment of BMI^(30)^. Furthermore, the test–retest reliability correlations for self-reports of height and weight in the EAT 2018 cohort were high (*r* = 0·98 in EAT 2018). Second, regarding meals, the quality, nutrient content and portion size of the food consumed at specific meals of the day were not assessed in this study. Relatedly, given that the context of meals and physical and social environments may play a crucial role in food choice trajectories^(10)^, future studies should explore the context of the meals and physical and social environments on meal consumption patterns and weight trajectories. Additionally, the participants were geographically restricted to the Minneapolis–St. Paul metropolitan area; thus, the findings might not be generalisable to other geographical regions. Because the number of participants who had a BMI less than the 15th percentile for their sex and age at baseline was relatively small (*n* 97), it is important to use caution when interpreting the results. Furthermore, the assessment of lunch and dinner intake was limited to only one time point in EAT 2010 and was not repeatedly measured in EAT 2018, which prevents the examination of changes in lunch and dinner intake over time and the examination of the relationship between these changes and BMI. Relatedly, all meals assessed in this study are referenced for the past week, which may not capture an individual’s comprehensive meal consumption pattern. Finally, as in all epidemiological studies, our study is limited by potential residual confounding from unmeasured variables.

Our findings regarding the association of regular consumption of breakfast, lunch and dinner with BMI trajectories from adolescence through emerging adulthood have several implications for researchers and clinicians. First, for clinicians, the lower the prevalence of regular consumption of meals among adolescents in the higher weight and highest weight categories, the positive associations with BMI among adolescents in the <15th percentile of sex- and age-specific BMI, and the positive but modest associations with BMI over 8 years of follow-up in the higher weight categories collectively illustrate that regular meal consumption might benefit the overall population, including persons across the BMI spectrum. Thus, the same recommendation – consistent meal consumption – may be applicable to the overall population of young people, regardless of their weight status.

For researchers, future studies should strive to identify factors that motivate or prevent adolescents from regularly consuming meals. For example, prospective population-based studies and experimental studies examining the content of meals as well as the context of meals (e.g. commensal eating, family meals, school lunch) and transition and turning points (e.g. change in home environment, attending college, marriage) on meal consumption routine and BMI would provide a more definitive level of evidence of the mechanism between meal intake and BMI trajectories during this transitional period between adolescence and young adulthood. Relatedly, future research should consider examining the role of appetite on regular consumption of meals and weight status, given that poor appetite could lead to frequent small meals and low weight status, while greater appetite may drive more frequent and regular meal consumption. Additionally, qualitative studies may further elucidate understanding the motivations for eating meals regularly as well as the barriers to regularly consume meal.

The potential and moderate findings of the associations of lunch and dinner consumption with BMI trajectory in the literature call for future observational studies to be replicated with participants in various age and racial/ethnic groups as well as across different weight categories, including larger groups of participants in the <15th percentile of sex- and age-specific BMI. Given the few studies that explore lunch and dinner consumption with BMI, additional studies are needed to replicate our analysis examining the associations of lunch and dinner with BMI. If replicated studies generate similar results, it may imply that healthcare professionals working with adolescents should discuss the broad range of benefits and risks associated with habitually eating meals on a regular basis.
